# Early Biocompatibility of Brown Macroalgal Scaffolds in a Splinted Full-Thickness Rodent Wound Model: A Pilot Study

**DOI:** 10.3390/jfb17080373

**Published:** 2026-08-01

**Authors:** Svava Kristinsdottir, Ottar Rolfsson, Olafur Eysteinn Sigurjonsson, Arni Kjalar Kristjansson, Hildur Sigurgrimsdottir, Jona Freysdottir, Sigurður Brynjolfsson, Sigrun Nanna Karlsdottir

**Affiliations:** 1 Industrial Engineering, Mechanical Engineering and Computer Science Department, University of Iceland, 102 Reykjavik, Iceland; sb@hi.is (S.B.); snk@hi.is (S.N.K.); 2 School of Health Sciences, Medical Department, University of Iceland, 102 Reykjavik, Iceland; ottarr@hi.is (O.R.); jf@hi.is (J.F.); 3 School of Science and Engineering, Reykjavik University, 102 Reykjavik, Iceland; oes@ru.is; 4 Department of Pathology, Landspitali-The National University Hospital of Iceland, 101 Reykjavik, Iceland; akjalar@hi.is; 5 Department of Immunology, Landspitali-The National University Hospital of Iceland, 101 Reykjavik, Iceland

**Keywords:** brown macroalgae, scaffold, wound healing, biocompatibility, biomaterials

## Abstract

Wounds are an increasing clinical and socioeconomic burden motivating the development of sustainable and biocompatible biomaterials for wound healing. Collagen-based extracellular matrix products can improve healing but are limited by cost and ethical or cultural constraints. Cellulose-rich macroalgal matrices offer a potential sustainable alternative. This pilot study evaluated the biocompatibility and wound-healing performance of brown macroalgal scaffolds. Scaffolds derived from *Laminaria digitata* (*LD*) and *Saccharina latissima* (*LS*) were produced using the visible-light method. Indirect cytotoxicity was assessed in keratinocytes, and in vivo effects were evaluated in a splinted dorsal excisional wound model in Sprague Dawley rats (*n* = 24), comparing *LD*, *LS*, bacterial cellulose (a CACS), and standard of care (SOC). The *LD* and *LS* extracts were non-cytotoxic, and no adverse reactions were observed in vivo. Wounds treated with *LD*, *LS*, and the SOC showed faster wound closure than the CACS at intermediate timepoints. Inflammation and foreign-body response scores were low and comparable across all groups. The *LD* showed significantly greater fibroblast infiltration than the CACS at day 7, and at day 14, the *LD* group exhibited the highest *collagen area fraction*. These findings indicate that *LD* and *LS* scaffolds are non-cytotoxic, are well-tolerated in vivo, and influence wound repair in full-thickness wound models, warranting further study as sustainable biomaterial.

## 1. Introduction

Wounds are a major healthcare issue, with the prevalence of chronic, non-healing wounds increasing worldwide [1,2,3]. This growing burden is largely driven by population aging and the rising prevalence of lifestyle-related diseases such as diabetes, obesity and vascular disease [2]. Diabetes can lead to neuropathy and impaired perfusion, thus increasing the risk of foot ulceration and subsequent lower-extremity amputation [4,5,6]. In addition to chronic wounds, acute wounds caused by trauma, surgery, and burns may be complicated by infection, excessive scarring, and functional impairment, particularly when tissue loss is extensive or healing is prolonged [7]. Appropriate wound care can promote tissue granulation and re-epithelialization. The use of collagen-based biological scaffolds for wound healing and tissue regeneration is well-documented and an effective treatment [8,9]. While collagen-based biological scaffolds have been shown to aid in wound healing, they have limitations due to high cost, immunological reactions, and cultural/religious concerns related to the human or animal origins of the scaffolds [10,11,12,13,14,15]. For those reasons, attention has shifted from collagen-based scaffolds toward the development and study of cellulose-based scaffolds, with promising results [16,17]. Cellulose is the most abundant biopolymer on Earth. It is the main structural component of terrestrial plant cell walls and many algae. It is also synthesized by a wide variety of bacteria and by tunicates (urochordates), the only animals known to synthesize cellulose [18]. Cellulose scaffolds have been developed from plants such as spinach, apples and celery and repopulated with mammalian cells, with good results [19,20]. Wound dressings that utilize the properties of cellulose are frequently formulated as composite materials, often incorporating nano-cellulose with chitosan, alginate or other polysaccharides. This combination improves fluid absorption, mechanical stability and bioactivity [21]. For example, highly porous cellulose sponges derived from Gleditsia triacanthos have shown promising wound-healing potential, pending in vivo validation [22]. Similarly, wood pulp-derived hydrogels and bacterial cellulose (BC) dressings have demonstrated comparable nanofibrillar structures, moisture handling, biocompatibility, and effective wound healing [22,23].

The biochemical, microstructural and mechanical characteristic properties of the brown-seaweed scaffolds derived from *Laminaria digitata (LD)* and *Saccharina latissima (Laminaria saccharina, LS)* have been described [24]. In brief, the *LD* and *LS* scaffolds are produced using a visible-light protocol designed to preserves the natural architecture of the macroalgae and produce a novice biocompatible scaffold [24]. Histological assessment has shown removal of visible cellular content while maintaining the overall extracellular matrix structure [24]. The resulting scaffold primarily consisted of complex carbohydrates, such as cellulose, alginate, fucoidan and amino acids [24]. Previously reported *LD* and *LS* scaffold properties that are relevant to wound application are summarized in Table 1. Briefly, scanning electron microscopy (SEM) demonstrated a heterogeneous, hierarchical structure comprising smaller chambers close to the surface and larger interconnected subsurface channels or tunnels closer to the center of the seaweed blade. This has resulted in a broad range of pore sizes, with internal chamber dimensions ranging from 46 to 149 µm, with the average pore size of *LD* being 48.9 ± 77.5 µm and of *LS* being 19.95 ± 17.72 µm. This difference in pore size reflects the pronounced structural heterogeneity of the natural macroalgal matrices. The micro-CT analysis revealed interconnected subsurface micro-tunnels with mean diameters of 81 ± 62 µm for *LD* and 55 ± 24 µm for *LS* [24]. Both scaffolds exhibited comparable porosity, of approximately 67%; high fluid absorption capacity values (>factor 10); and hydrated tensile properties within a soft tissue-relevant range [24]. In combination with the surface micropores, this architecture falls within scaffold design features reported to favor cellular ingrowth and support wound healing [24,25]. The use of *LD* and *LS* as structurally intact biological scaffolds is largely an unexplored field, although recently published research is promising for a variety of seaweed types [26,27,28].

The primary objective of this pilot study was to assess and compare, in a full-thickness wound model, the biocompatibility and wound-healing effects of *LD* and *LS* scaffolds in comparison to a commercially available cellulose sheet (CACS, EPIPROTECT^®^) and standard of care (SOC, Tegaderm^®^). Early-stage evaluation of novel biomaterials often begins with full-thickness small-animal wound models to assess local tolerance and early wound-healing responses [29]. Rodent wounds heal predominantly through contraction, but that can be minimized by using a splinted wound model to promote granulation tissue formation and re-epithelialization [29,30]. To this end, a splinted full-thickness rat wound model was employed. The key endpoints used to assess biocompatibility and wound healing included immunological response, wound closure rate, and the neo-tissue index (NTI), defined as the ratio between the thickness of repair tissue in the wound bed and that of adjacent unwounded skin on the same section providing relative tissue thickness.

We hypothesized that intact brown-seaweed scaffolds derived from *LD* and *LS* would be non-cytotoxic and elicit an in vivo immunological response comparable to the CACS and SOC while supporting granulation, re-epithelialization and tissue restoration in full-thickness splinted wounds.

## 2. Materials and Methods

### 2.1. LD and LS Scaffold Preparation

The *LD* and *LS* scaffolds were prepared at the University of Iceland according to the protocol described in a previously published article [24]. In short, the seaweed *LD* and *LS* were collected along the Reykjanes Peninsula coastline, Iceland, in autumn of 2023 (GPS: 63.97056885259877, −22.75364300250477). The collected seaweed was rinsed with cool water, sorted and frozen at −14 to −20 °C for storage. Next, the thawed *L.D* and *L.S* seaweed samples were mounted into frames and placed into tanks with continuous waterflow at <12 °C with compressed air to produce turbulence in the plastic see-through tanks. Light panels were positioned on the opposite sides of the tanks, emitting electromagnetic radiation at 620 nm and 470 nm for 10 days each to decellularize the seaweed. Following the decellularization of the *LD* and *LS* samples, the seaweed scaffolds were lyophilized at −50 °C and under a pressure of 0.5 pascal [24]. Finally, the *LD* and *LS* scaffolds were cut into 3 cm× 7 cm pieces, packaged using Steriking^®^ LT-Blueline Tyvek^®^ sterilization roll material (LTR41, Wipak Oy, Nastola, Finland), sealed into pouches and sterilized using vaporized hydrogen peroxide.

### 2.2. Indirect Cytotoxicity

Cytotoxicity tests using the alamarBlue assay (AB) were performed according to ISO 10993-5 and ISO 10993-12 standards to evaluate *LD* and *LS* scaffold media extract effects on keratinocytes [31,32]. AB is a generally non-cytotoxic, cell-permeable viability reagent containing resazurin, a blue, weakly fluorescent redox indicator. In metabolically active cells, resazurin is reduced to resorufin, which is pink and highly fluorescent. The measured fluorescence or absorbance signal increases with cellular metabolic activity and the number of viable cells, thereby providing an indirect measure of cell viability.

The media extract was prepared per ISO 10993-12 guidelines for porous materials. Sterilized *LD* and *LS* scaffolds were incubated in a culture medium (DMEM with growth media) at 37 °C, 5% CO_2_ for 24 h, with shaking at 20 rpm for 10 min five times during incubation to produce the extract. The scaffold-soaked media was extracted and filtered through 0.22 µm filters to remove large scaffold fragments that could have interfered with subsequent measurements.

HaCaT human immortalized keratinocytes (CLS, catalog no. 300493; CVCL_0038; RRID) were evaluated using three independent cell passages. For each passage, cells were seeded in triplicate at a density of 1 × 10^4^ cells per well in 96-well plates and incubated overnight. The cells were then incubated with 100%, 50% and 30% concentrations of media extracts for 72 h. The absorbance measurements were taken after 4 h of incubation with 10% AB solution at the start of the test (t = 0) and then after 24, 48 and 72 h of incubation (t = 24, 48, 72). For comparison, cells cultured with regular media, blank media and 10% AB solution in media and a cytotoxic control of 70% methanol in media were included as controls.

The percentages of reduction in AB absorption for all groups were calculated using the AB percentage difference equation as per industrial instructions from Bio-Rad [33]:

(1) $$\mathrm{Percentage}\;\mathrm{difference}\;\frac{\left(O2\times A1\right)-\left(O1\times A2\right)}{\left(O2\times P1\right)-\left(O1\times P2\right)}\times 100$$

where *O*1 and *O*2 represent each molar extinction coefficient (E) of the oxidized AB at 570 and 600 nm, respectively. *A*1 and *A*2 represent the absorbance values of the test wells at 570 and 600 nm, respectively. *P*1 and *P*2 represent the absorbance values of the control well with no test agent (cells with normal media and AB solution but no test agent, i.e., 0% extract) at 570 nm and 600 nm, respectively.

### 2.3. Full-Thickness Wound Rat Model

This study was designed as an exploratory pilot study, approved by the Icelandic Food and Veterinary Authority (MAST) on 12 September 2023 (Ref. no. 2307723), and conducted in accordance with Icelandic legislation and the institutional guidelines for the care and use of laboratory animals. Twenty-four young male Sprague Dawley rats (weighing 250–300 g) were included to provide eight animals at each of three predefined assessment timepoints: day 7, day 11 and day 14. This number was considered sufficient to support comparisons among treatments while minimizing animal use in accordance with the principles of Replacement, Reduction, and Refinement.

The experimental groups consisted of the following four treatment categories: the seaweed scaffolds *LD* and *LS*, a commercially available cellulose sheet (CACS) from bacterial cellulose (EPIPROTECT^®^), and a standard of care (SOC).The secondary dressing for the *LD*, *LS*, and CACS groups was Tegaderm^®^ transparent film dressing (10 cm × 12 cm, catalog no. 1616, 3M Health Care, St. Paul, MN, USA), while the SOC group was covered exclusively with the Tegaderm^®^ film.

Each rat received four dorsal full-thickness wounds (8 mm), and each wound received one treatment of *LD*, *LS*, CACS or SOC, randomly assigned. Silicone rings were glued and sutured around each wound to minimize contraction and promote granulation and epithelialization, following the methodology established by Galiano et al. and adapted to this rat study [34].

Post-surgical procedures included placing the animals in a preheated recovery area and monitoring them until full ambulation was regained. Daily observations of the animals’ general wellness were conducted, with individualized evaluations performed for any abnormal behavior or potential complications, including infection. Antibiotics and pain medication were administered according to approved protocol.

Euthanasia was carried out at predetermined time intervals: day 7, day 11, and day 14. At each timepoint, images of the wounds were taken, and tissue samples were collected for subsequent analysis. This study protocol adhered strictly to ethical guidelines, ensuring the welfare of the animals throughout the experimental timeline.

### 2.4. Wound Closure Rate

The wounds of each rat were photographed under general anesthesia (isoflurane) at the designated endpoint (day 7, 11, or 14), and the wound area subsequently measured using ImageJ software (ImageJ 1.53k). The mean of three independent measurements was used to calculate the percentage of wound closure using Equation (2):

(2) $$Wound\;Closure\;\left(\%\right)=\left[\frac{\left(A_{initial}-A_{day\;x}\;\right)}{A_{initial}}\right]\;\times 100$$

where *A_initial_* is the initial wound area and *A_day x_* is the wound area at day 7, 11, or 14.

### 2.5. Histological Evaluation Using Semi-Quantitative Assessments on H&E-Stained Tissue Samples

Histological samples were collected post-euthanasia using a biopsy tool to excise the wound site. The specimens were fixed in 4% formalin for preservation. Standard histological processing was conducted, including paraffin embedding and sectioning, followed by staining with hematoxylin and eosin (H&E) and Masson trichrome (MT) for microscopic evaluation.

A blinded dermatopathologist conducted semi-quantitative histological assessments on H&E samples. The semi-quantitative analysis comprised assessment of the inflammatory response, foreign-body reaction, fibroblast infiltration, and revascularization using a scoring system ranging from 1 (low) to 2 (moderate) to 3 (marked), with a provision for half-point increments.

### 2.6. Quantification of Collagen Deposition via Collagen Area Fraction Measurement

Brightfield images were acquired at 40× magnification using a light microscope (T720; AmScope, Irvine, CA, USA) equipped with a digital camera (MU1803; AmScope, Irvine, CA, USA) from tissue samples collected on days 11 and 14 and stained with Masson’s trichrome (MT). For each image, a region of interest (ROI) was manually delineated at the wound edge beneath the newly formed epidermis to include granulation tissue while excluding non-representative areas and obvious artifacts, such as folds, tears, and debris.

To quantify collagen deposition, color deconvolution was performed to separate the individual stain components using the Fiji software (ImageJ 1.54p). The collagen channel was identified based on the blue MT signal. Collagen-positive pixels within the predefined ROI were then segmented by thresholding using parameters optimized against the original microscope images. The collagen area fraction was calculated as

(3) $$Collagen\;area\;fraction\;\%=\left(\frac{N_{ROI\;collagen\;}}{N_{ROI}}\right)\;\times 100$$

where *N_ROI collagen_* is the number of Masson’s trichrome-positive pixels within the ROI and *N_ROI_* is the total number of pixels within the ROI. For each wound, values from two 40× fields were averaged to obtain a single collagen-area fraction value. Tissue samples from day 7 were not analyzed, as the collagen deposition was considered premature for meaningful assessment at that timepoint. All image analyses were performed blinded.

### 2.7. Neo-Tissue Index Definition and Measurements

The neo-tissue index (NTI) was calculated according to Equation (4):

(4) $$NTI\;\left(\%\right)=\frac{T_{neo}}{T_{adjacent}}\times 100$$

where *T_neo_* represents the full thickness of the neo-tissue, measured from the adipose tissue to the outer edge of the epidermis, and *T_adjacent_* represents the thickness of the adjacent uninjured skin measured using the same boundaries. Images of hematoxylin and eosin (H&E)-stained tissue sections at 10× magnification were taken and analyzed using ImageJ software 1.54g. For each specimen, three independent measurements were obtained for both neo-tissue thickness and adjacent uninjured skin thickness in a blinded manner. The median neo-tissue thickness was then divided by the median adjacent skin thickness to calculate the NTI. By definition, an NTI of approximately 100 indicates tissue thickness comparable with that of adjacent normal skin, whereas values below 100 indicate thinner repair tissue and values above 100 indicate thicker repair tissue. When present, hair follicles and sebaceous glands were avoided as anatomical landmarks to minimize bias.

The NTI was used as a composite internal-reference morphometric index of overall repair-tissue thickness rather than as a compartment-specific measure of dermal thickness alone. This approach was selected to provide a simple and reproducible comparative measure of wound-bed thickness that was less dependent on the precise delineation of epidermal and dermal boundaries across healing stages and potentially less sensitive to variation introduced by local wound geometry or sectioning orientation. The NTI was therefore interpreted as a complementary structural endpoint alongside more compartment-informative histological outcomes, including epithelial continuity, fibroblast infiltration, and collagen deposition.

### 2.8. Statistical Analysis

For cytotoxicity testing, differences between groups at each timepoint were assessed using one-way analysis of variance (ANOVA), followed by Tukey’s post hoc test for pairwise comparisons. In vivo outcomes were primarily analyzed using mixed-effects models with RatID as a random intercept to account for within-animal clustering. For the semi-quantitative histological scores, sensitivity analyses were additionally conducted using cumulative link mixed models (CLMMs). For continuous outcomes, including healing rate and collagen area fraction, Tukey-adjusted post hoc pairwise comparisons were performed following the overall model to identify differences between individual groups. Statistical significance was set at *p* < 0.05. All statistical analyses were performed using R version 4.5.0 (R Foundation for Statistical Computing, Vienna, Austria) within RStudio version 2026.01.0+392 “Apple Blossom” (Posit Software, PBC, Boston, MA, USA).

### 2.9. Exclusion Criteria

Histological slides and images were evaluated blindly. Any slides or images deemed to be of insufficient quality to the extent that they impaired accurate assessment and measurements were excluded from the data analysis process to ensure the integrity and reliability of the results.

## 3. Results

### 3.1. In Vitro Testing Indicates That the LD and LS Scaffolds Are Non-Cytotoxic

To assess the potential cytotoxicity of the *LD* and *LS* scaffolds, indirect cytotoxic testing was performed in accordance with ISO-10993-12 and -5 standards for medical devices. Both scaffold types were evaluated using the indirect media-extract method in cultured keratinocytes.

**Figure 1:**
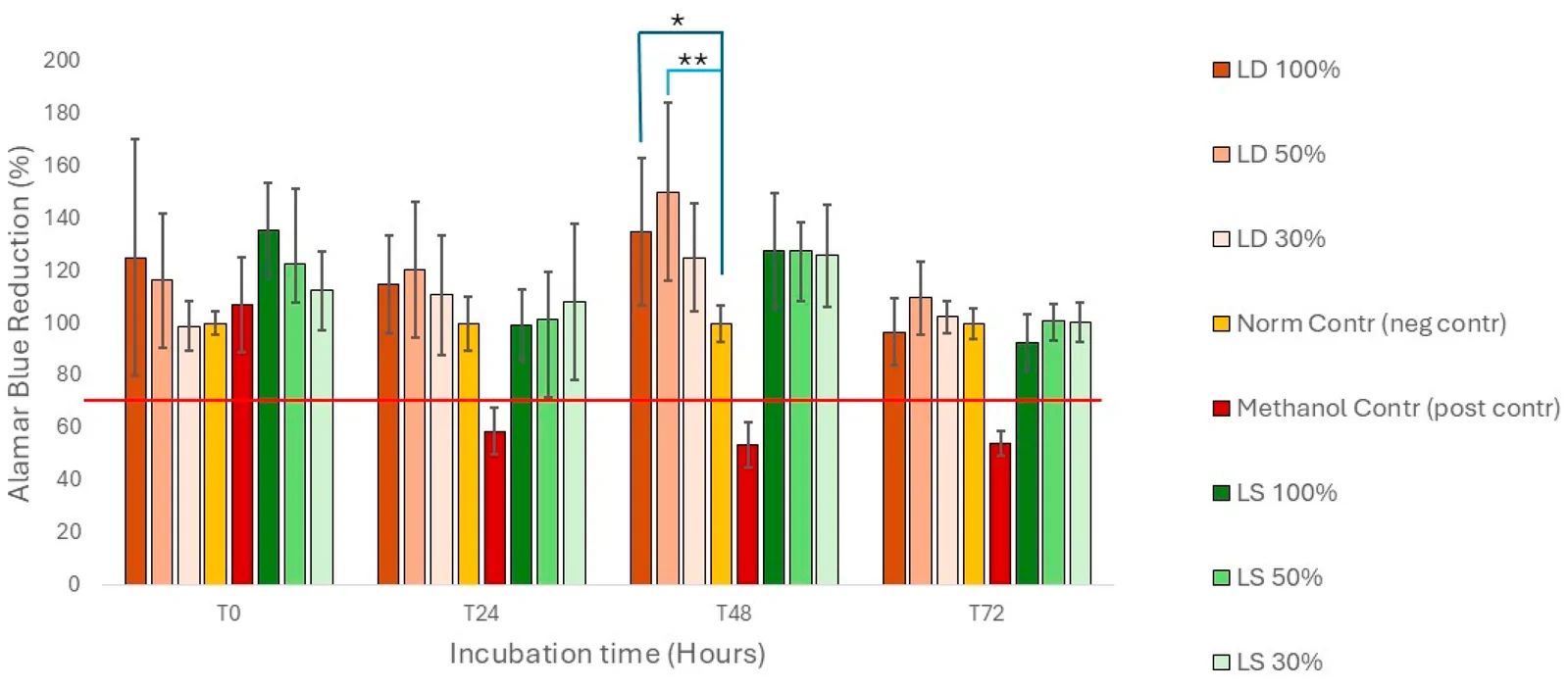
Indirect cytotoxicity assessment of *LD* and *LS* scaffolds using an alamarBlue assay. Indirect cytotoxicity testing was performed in accordance with ISO-10993-12 and ISO-10993-5 using media extracts from *LD* and *LS* brown-seaweed scaffolds. Keratinocytes were incubated with 100%, 50%, and 30% scaffold extracts for 24, 48, and 72 h, and cell viability was quantified using the alamarBlue assay. Negative controls consisted of cells incubated with standard culture media, while 70% methanol served as a positive cytotoxic control. A red solidline indicates the 70% viability threshold distinguishing non-cytotoxic from cytotoxic conditions per ISO-10993 guidelines. Across all timepoints, all *LD* and *LS* extract conditions exhibited significantly higher cell viability than the methanol control. No significant differences were observed between the *LD* or *LS* extract and the negative control at 24 h. At 48 h, the *LD*-100% and *LD*-50% extracts showed significantly higher viability compared to the negative control. No significant differences between the *LD* or *LS* extract and the negative control were detected at 72 h. Values are presented as means ± SD (n = 9); * *p* < 0.05, ** *p* < 0.01.

The AB reduction measurements showed similar cell activity at the start of the experiment (T = 0) for all groups (*p* > 0.05) (Figure 1). All *LD* and *LS* extract conditions demonstrated AB reduction values exceeding 70% at all measured timepoints, surpassing the methanol control. Across all timepoints (24, 48, and 72 h), each experimental group exhibited significantly greater AB absorption compared to the methanol control. No significant differences between *LD* or *LS* and the normal control group were noted at T24. By T48, significant differences emerged between *LD*-100% (*p* = 0.02) and *LD*-50% (*p* = 0.0004) versus the normal control group. No significant differences were detected between the *LD* or *LS* group and the normal control group at T72.

At every timepoint, the mean AB reduction in all *LD* and *LS* groups consistently surpassed the 70% threshold specified by ISO-10993, confirming that the extracts did not induce cytotoxic effects in cultured keratinocytes (Figure 1).

### 3.2. No Treatment-Related Adverse Events Were Observed During Animal Study

The rats exhibited good tolerance to the procedures, with minimal complications observed and prompt recovery following general anesthesia. Wound treatments were applied directly to the wound bed and were only replaced if dislodged or removed by the animal. Instances of silicone splint removal by the animals were observed, leading to periods of un-splinted wounds. In such cases, the silicone splints were reapplied promptly upon discovery. Two instances required the reapplication of *LD* scaffolds due to displacement caused by animal behavior. One animal died during anesthesia while undergoing reapplication of the silicone splint on day 5, resulting in exclusion from this study. Throughout the study period, none of the animals exhibited adverse reactions related to the wound treatment or showed signs of infection.

### 3.3. Wounds Managed with LD and LS Scaffolds Exhibited Faster Closure Relative to CACS at Intermediate Timepoints: Groups Converged by Day 14

**Figure 2:**
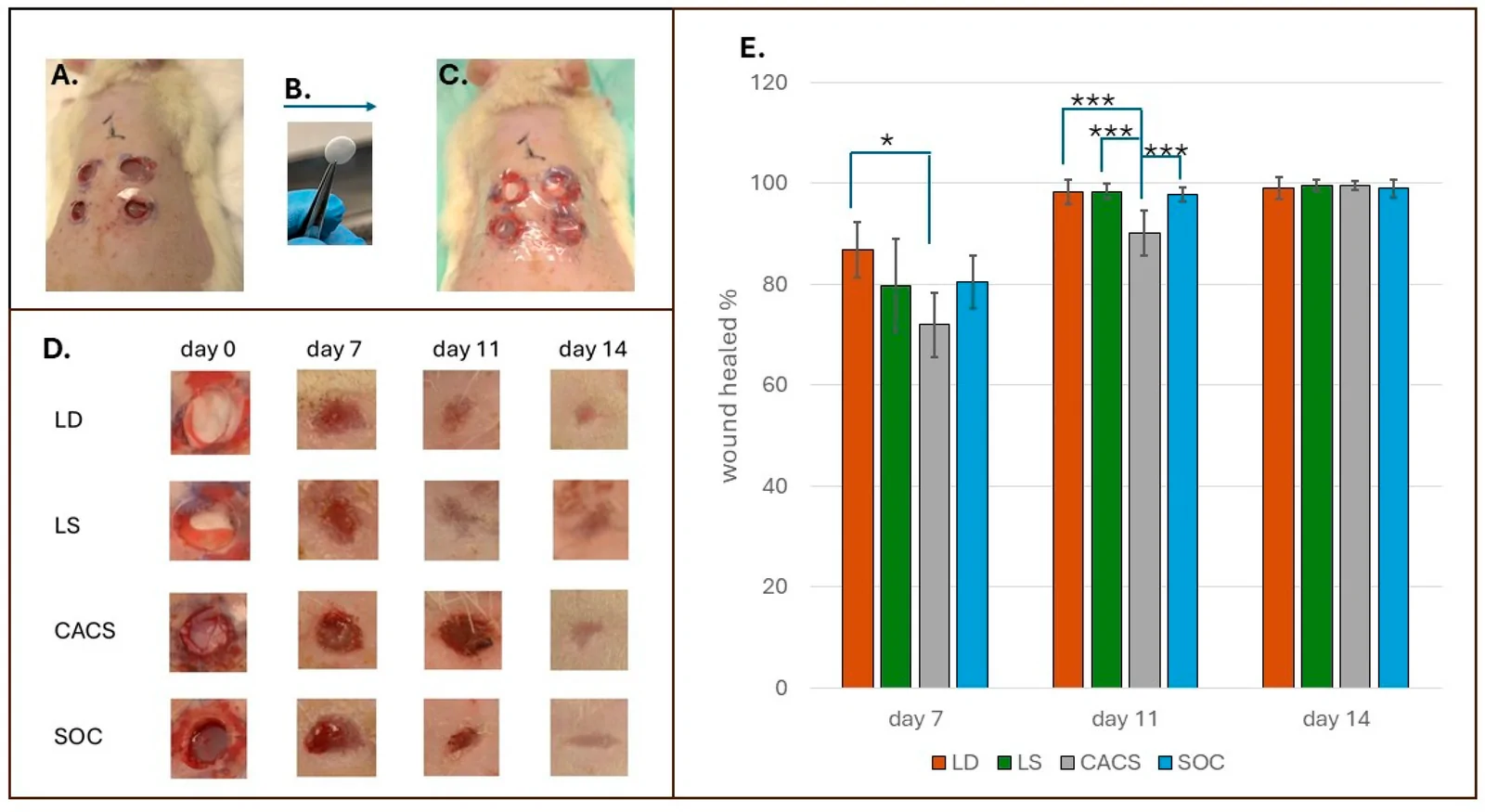
Wound closure progression following treatment with scaffolds and controls. (**A**) Representative photographs of four full-thickness dorsal excisional wounds created on the back. (**B**) Photograph of an *LD* scaffold prior to rehydration. (**C**) Representative image after application of silicone splints to minimize wound contraction; splints were secured using adhesive and sutures, and wounds were treated with *LD* scaffold, *LS* scaffold, CACS, or SOC, with Tegaderm used as a secondary dressing. (**D**) Representative wound photographs for each treatment group at baseline (day 0) and on days 7, 11, and 14. (**E**) Quantification of wound closure (% wound healed) on day 7, day 11 and day 14, presented as means ± standard deviation. Wound area was measured in a blinded manner using ImageJ. At day 7, wound closure was significantly greater in the *LD* group compared to CACS, and by day 11, CACS remained significantly lower than *LD*, *LS*, and SOC; by day 14, wound closure converged across all groups, with no significant differences. Statistical significance is indicated by asterisks in the graph (* *p* < 0.05, *** *p* < 0.001).

The wound-healing rate was assessed by measuring the area healed, with 100% indicating complete closure and no remaining wound area. Images of the wounds were captured on days 7, 11, and 14, and the wound areas were measured quantitatively in a blinded manner using ImageJ software (Figure 2A–E).

Across timepoints, wound closure differed between groups early on, but they converged by day 14; see Figure 2. At day 7, the mean ± SD closure was highest in *LD* (87.1 ± 4.76%, n = 5), followed by SOC (80.4 ± 5.25%, n = 4) and LS (79.8 ± 9.26%, n = 4), while CACS showed the lowest closure (71.9 ± 6.34%, n = 4); a mixed-effects model showed an overall group effect (χ^2^(3) = 12.60, *p* = 0.0056), with *LD* significantly higher than CACS in Tukey-adjusted post hoc testing ( *p* = 0.031). By day 11, closure was near complete in *LD*, *LS*, and SOC (LD—98.3 ± 2.44%, n = 6; *LS—*98.3 ± 1.60%, n = 7; SOC—97.7 ± 1.32%, n = 6), whereas CACS remained lower (90.0 ± 4.43%, n = 6); the mixed-effects model again indicated a strong overall group effect (χ^2^(3) = 65.31, *p* = 4.3 × 10^−14^), and Tukey-adjusted comparisons showed that CACS was significantly lower than *LD*, *LS*, and SOC (all *p* ≤ 0.0001), with no differences among *LD*, *LS*, and SOC. By day 14, all treatments achieved near-complete closure (*LD—*99.0 ± 2.19%, *LS*—99.5 ± 1.11%, CACS—99.5 ± 0.96%, SOC—98.9 ± 1.82%; n = 5), and there was no overall group effect (χ^2^(3) = 0.99, *p* = 0.803) and no significant pairwise differences, indicating convergence of closure outcomes at the final timepoint.

### 3.4. NTI in LD and LS Groups Trended Closer to Adjacent Unwounded Skin at Day 14

**Figure 3:**
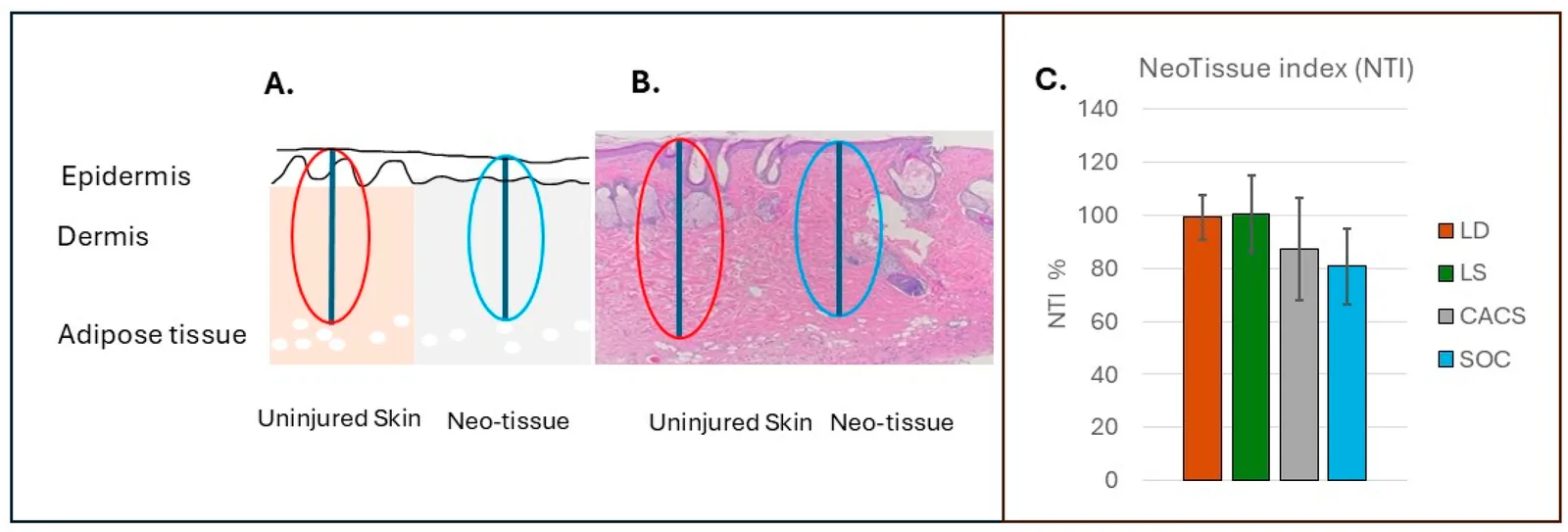
Assessment of neo-tissue thickness at day 14. (**A**) Schematic illustration of the measurement approach. The thickness of the adjacent uninjured skin was measured vertically from the epidermal surface to the boundary of the adipose tissue, as indicated by the red ellipse and dark-blue line. Neo-tissue thickness was measured in the same manner within the wound area, as indicated by the light-blue ellipse and dark-blue line. (**B**) Representative histological section showing superimposed measurement lines used for thickness calculations. (**C**) NTIs quantified at day 14 across four treatment groups: *LD*, *LS*, CACS, and SOC. NTI values were highest in the seaweed scaffold groups (*LD* and *LS*) and lower in the control groups, presented as means ± standard deviation. This difference was not statistically significant.

At day 14, the NTI was highest in the seaweed scaffold groups and lower in the control groups (*LD*—99.3 ± 8.5%, n = 6; *LS*—100.0 ± 14.6%, n = 6; CACS—87.3 ± 19.4%, n = 6; SOC—80.7 ± 14.5%, n = 7) (Figure 3). A linear mixed-effects model with group as a fixed effect and RatID as a random intercept demonstrated an overall treatment effect on the NTI (Type III Wald χ^2^(3) = 9.25, *p* = 0.026). In the model’s fixed effects (reference = *LD*), the SOC group showed a significantly lower NTI than *LD* (estimate = −0.183, *p* = 0.028), whereas *LS* and CACS did not differ significantly from *LD*. The estimated marginal means (EMMs) were as follows: *LD—*0.990 (95% CI: 0.864–1.116), *LS—*0.999 (0.873–1.125), CACS—0.869 (0.743–0.995), and SOC—0.807 (0.691–0.923). However, Tukey-adjusted pairwise comparisons did not identify significant differences (all adjusted to *p* ≥ 0.095), with the closest contrast being *LS* vs. SOC (adjusted *p* = 0.095).

### 3.5. LD-Treated Wounds Showed Greater Early Fibroblast Infiltration than CACS, with No Significant Between-Group Differences in Inflammation or Foreign-Body Response

**Figure 4:**
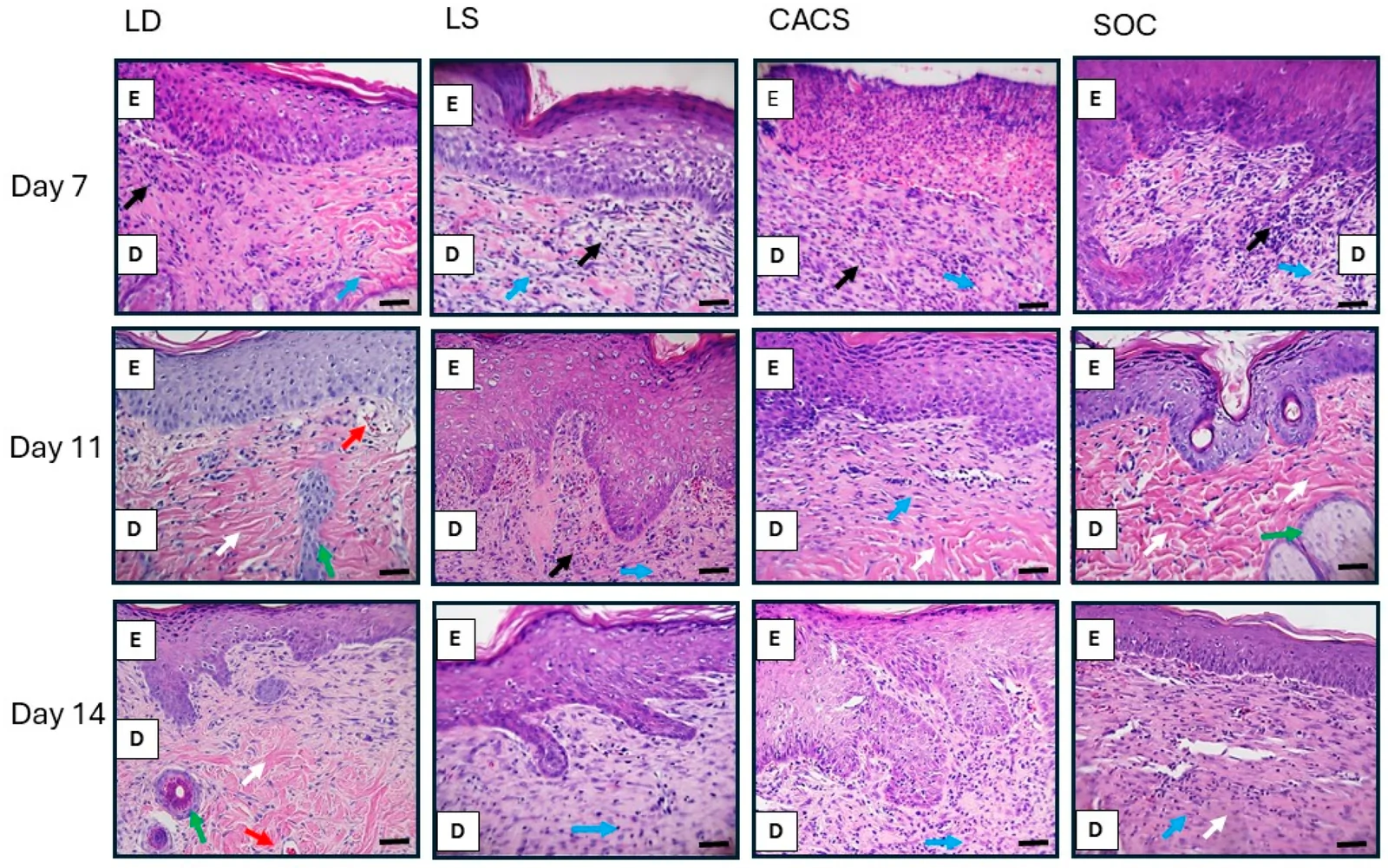
Representative histological appearances across treatment groups and timepoints. Hematoxylin and eosin (H&E)–stained tissue sections from *LD*, *LS*, CACS, and standard of care (SOC) treatment groups at days 7, 11, and 14 following euthanasia. Histological evaluation was performed in a blinded manner by a pathodermatologist to assess inflammation, foreign-body reaction, re-vascularization, and fibroblast infiltration. In H&E staining, hematoxylin stains cell nuclei dark blue to purple, while eosin stains the cytoplasm and extracellular matrix pink. Colored arrows indicate representative features: red—blood vessels; black—neutrophils; blue—fibroblasts; white—collagen bundles; and green—adnexal structures (hair follicles or sebaceous units). E, epidermis; D, dermis. Images were acquired using a 40× objective. Scale bar = 50 µm.

Histological evaluations and semi-quantitative scoring of H&E-stained tissue sections were performed in a blinded manner by a pathodermatologist following rat euthanasia on days 7, 11, and 14. On day 7, wounds showed incomplete to immature re-epithelialization, prominent inflammatory infiltrates, and fibroblast-rich granulation tissue within the dermis. By day 11, the epidermis appeared more continuous and differentiated, while the dermis showed increased fibroplasia, neovascularization, and collagen deposition. At day 14, most groups exhibited a more organized dermal matrix with reduced inflammatory cellularity, consistent with transition into the remodeling phase. In selected sections, adnexal structures such as hair follicle profiles were observed, suggesting more advanced tissue restoration (Figure 4).

**Figure 5:**
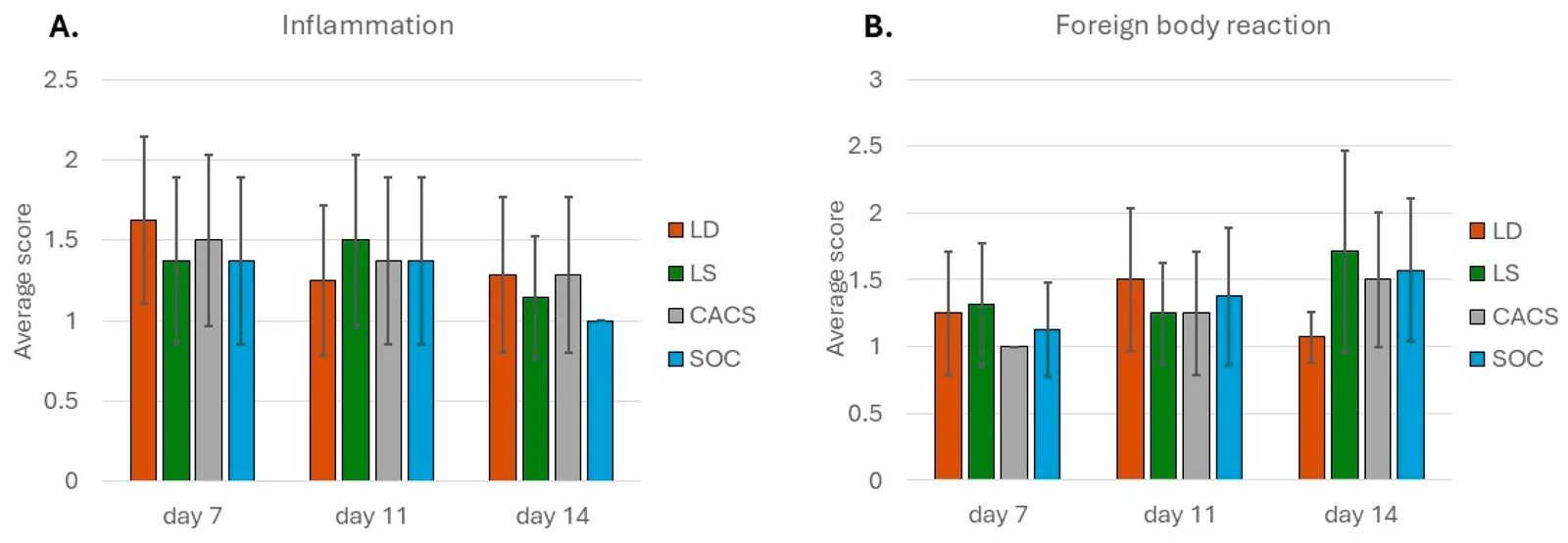
Histological assessment of inflammation and foreign-body responses over time. Semi-quantitative histological scoring was performed to assess inflammation and foreign-body responses in the four treatment groups (*LD*, *LS*, CACS, and SOC) at days 7, 11, and 14. Scores reflect evaluation of inflammation and foreign-body responses using the scoring system of 1 (low) to 2 (moderate) to 3 (marked), with a provision for half-point increments. (**A**) The inflammation scores werelow overall and comparable across groups at all timepoints, with no statistically significant differences. (**B**) The foreign-body response scores similarly remained low across groups and timepoints, with no statistically significant differences. Results are presented as mean ± standard deviation.

**Figure 6:**
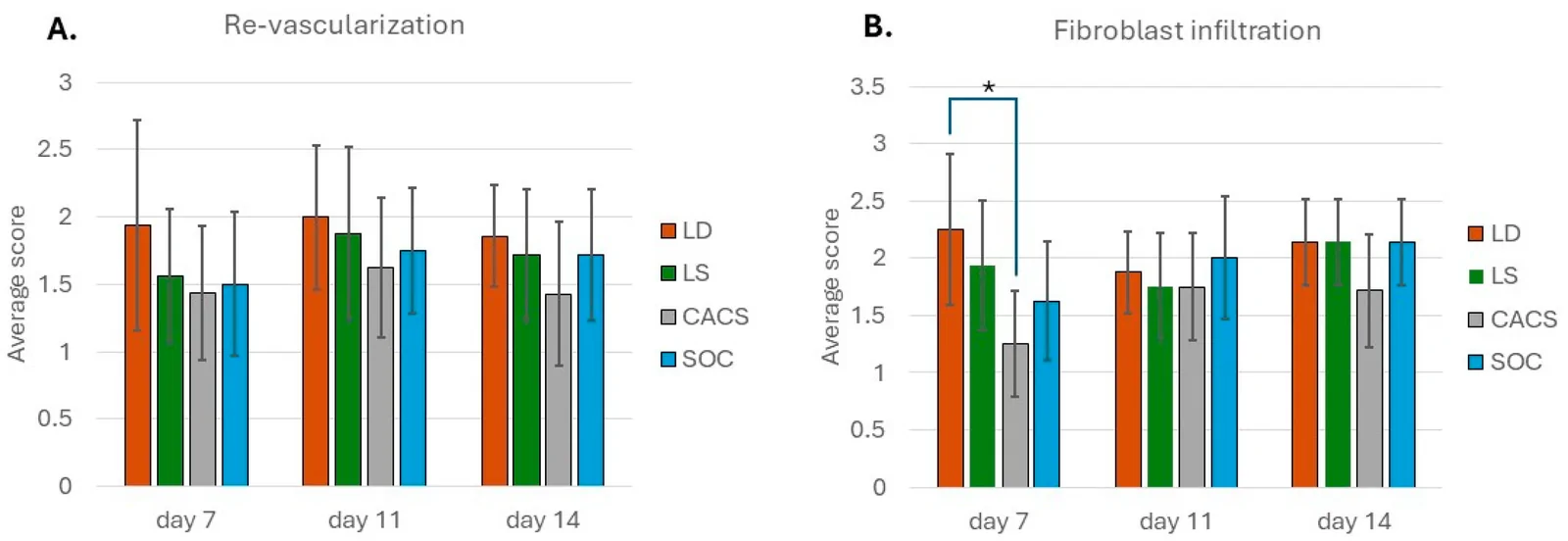
Semi-quantitative histological assessment of re-vascularization and fibroblast infiltration over time. Revascularization and fibroblast infiltration were scored in four treatment groups (*LD*, *LS*, CACS, and SOC) at days 7, 11, and 14 using the scoring system of 1 (low) to 2 (moderate) to 3 (marked), with a provision for half-point increments, here presented as means ± standard deviation. (**A**) Revascularization scores did not differ significantly between groups at any timepoint. (**B**) Fibroblast infiltration score differed by treatment at day 7, with higher scores in the *LD* group compared to CACS (χ^2^(3) = 13.67, *p* = 0.0034), but no significant group differences couldbe observed at days 11 or 14. Results are presented as mean ± standard deviation. Asterisks denote statistical significance (* *p* < 0.05).

Semi-quantitative histological assessments found the inflammation, foreign-body reaction and re-vascularization scores to be broadly similar across groups and timepoints with no significant difference (Figure 5 and Figure 6A). Fibroblast infiltration score was, however, significantly higher in the *LD* group on day 7 in comparison to the CACS group(Figure 6B). The mean inflammation score at all timepoints showed no statistically significant differences between the test groups (day 7: *p* = 0.72; day 11: *p* = 0.81; day 14: *p* = 0.47). Foreign-body response scores were also low across the groups at all timepoints (day 7: *p* = 0.18; day 11: *p* = 0.64; day 14: *p* = 0.13). No differences were detected in Tukey-adjusted pairwise analysis in both cases.

Revascularization scores were similar across the groups at all timepoints, and no statistical differences were noted across the timepoints (day 7: *p* = 0.32; day 11: *p* = 0.44; day 14: *p* = 0.39). The fibroblast infiltration scores were significantly higher in the *LD* group than in the CACS group on day 7 (2.12 ± 0.84 vs. 1.25 ± 0.46), according to the mixed-effects model (*p* = 0.0034). Sensitivity analysis using a cumulative-link mixed model yielded consistent results (likelihood ratio test, *p* = 0.0069; *LD* vs. CACS, *p* = 0.034). By day 11, fibroblast infiltration did not differ between groups (*p* = 0.548). By day 14, the scores increased across all treatment groups, but the between-group differences remained non-significant (*p* = 0.400).

### 3.6. Collagen Area Fraction Was Significantly Higher in the LD Scaffold Group at the Final Timepoint

**Figure 7:**
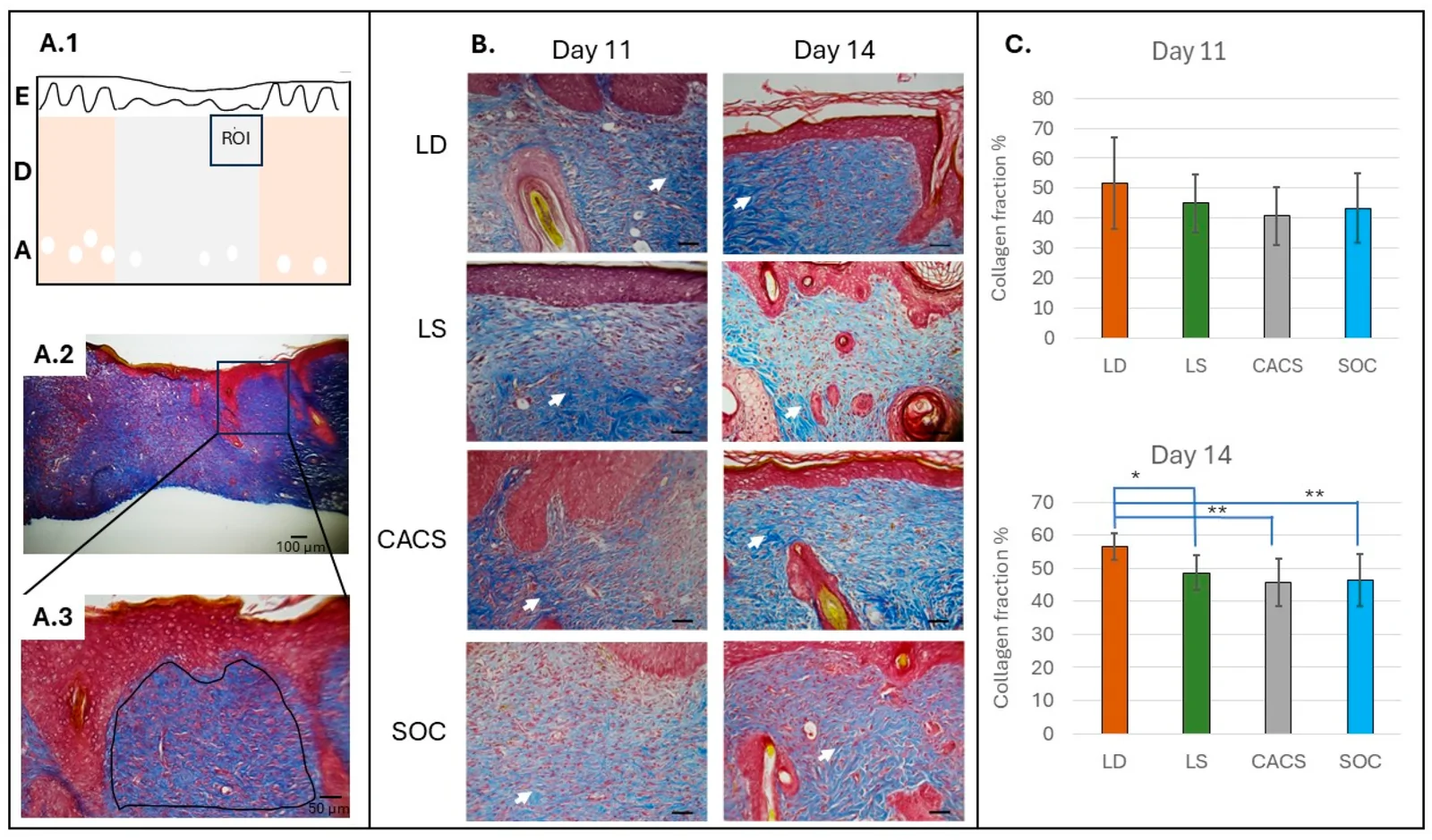
Quantification via collagen area fraction (%). (**A.1**) Schematic illustration depicting the definition of the region of interest (ROI): E—epidermis, D—dermis, A—adipose tissue. (**A.2**) Representative histological images depicting the ROI at 10× magnification, with a scale bar of 100 µm. (**A.3**) A higher-magnification view from the 10× image, showing the delineated ROI on representative histological sections at 40× magnification; scale bar = 50 µm. (**B**) Representative MT-stained wound-edges for each treatment group (LD, LS, CACS, SOC) at day 11 and day 14; scale bar = 50 µm. (**C**) At day 11, the collagen area fraction did not differ significantly among groups. At day 14, the *LD*-treated wounds exhibited a higher collagen area fraction compared to *LS*, CACS, and SOC, while no significant differences were observed between *LS*, CACS, and SOC. Data are presented as means ± standard deviation. Statistical significance is indicated by asterisks in the graph (* *p* < 0.05, ** *p* < 0.001).

Collagen deposition was assessed by calculating the collagen area fraction (%) in ROIs from MT-stained tissue samples from day 11 and 14, following euthanasia. Calculation of the collagen area fraction did not show statistically significant differences between groups on day 11 (*p* = 0.0798). However, on day 14, the collagen area fraction differed significantly among the groups (Figure 7). Here, statistical analysis demonstrates a significant overall group effect (*p* = 0.00020). Tukey-adjusted post hoc testing showed that *LD* had a higher collagen area fraction compared to LS (*p* = 0.0328), CACS (*p* = 0.0064), and SOC (*p* = 0.0119). No significant differences were found between the LS, CACS, and SOC groups (adjusted *p* ≥ 0.776).

## 4. Discussion

This study provides early evidence that brown-seaweed scaffolds are biocompatible in vitro and in vivo, supported by ISO-10993 extract cytotoxicity testing and histological evaluation in a splinted full-thickness rodent wound model.

### 4.1. Cytotoxicity and Immunological Responses of the LD and LS Scaffolds

To assess cytotoxicity, the ISO 10993 indirect extract assay was used to determine whether the substances released from the *LD* and *LS* scaffolds reduced cellular metabolic activity below the cytotoxicity thresholds. Although this approach is sensitive to leachable-mediated toxicity, it does not capture cell–material interactions that depend on biomaterial architecture [35,36]. To complement these assays, an in vivo full-thickness rat wound model, which better reflects the biological complexity of wound healing, including immune responses, enzymatic remodeling, and mechanical stresses, was used [37,38]. Within this model, histological assessment of the immunological response included inflammation and foreign-body reaction, neither of which differed significantly between groups at any timepoint. In addition, no adverse wound reactions or signs of infection were observed during this study. In vivo biocompatibility of green-seaweed scaffolds in an implant rodent model has supported the broader premise that macroalgae-derived scaffolds are tolerated in vivo [39]. While implantation models are not directly equivalent to full-thickness wound models, they provide complementary evidence that seaweed-derived scaffolds can be compatible with living tissue.

Collectively, these findings indicate that the *LD* and *LS* scaffolds do not provoke an exaggerated local host response and were broadly comparable to CACS and SOC in terms of tissue compatibility within the study period. However, as this was a pilot study, the findings should be interpreted cautiously, and further work using larger cohorts and more detailed immunological characterization will be needed to better understand the interactions between these novice biomaterials and the biological host.

### 4.2. Early Wound Healing and Tissue Restoration with LD and LS Seaweed Scaffolds

Rapid and complete wound closure is a key objective of skin repair, as it helps restore barrier function and reduce the risk of infection and further tissue injury [40]. In this study, all treatment groups achieved wound closure by day 14; however, statistically significant differences between the groups were observed at the initial two timepoints. On day 7, the percentage of wound closures was significantly lower in the CACS group than in the *LD* group. On day 11, the wound closure percentage in the CACS group was significantly lower than in the *LD*, *LS*, and SOC groups. By day 14, all wounds were considered closed in all groups and no statistical differences were found between the groups. Rodent excisional wound models are widely used for preclinical screening wound dressings and biomaterials, but interpretation depends strongly on model design because rodents heal largely by contraction unless it is mechanically limited (e.g., splinting). Reviews of rat wound models highlight that splinting improves translational relevance by shifting healing toward re-epithelialization and granulation, but variability can be introduced if splints are displaced or if wound edges experience intermittent contraction [41]. In the current pilot study, splint loss and reapplication episodes may therefore have contributed to the between-animal variance in closure kinetics and should be controlled more strictly in future studies.

To further assess the structural restoration of the healed wound bed, the NTI was introduced, defined as the ratio of the thickness of newly formed tissue to that of adjacent unwounded skin on the same section, conceptually similar to the scar evaluation index (SEI) [42]. Unlike dedicated hypertrophic scar models such as the rabbit ear or rat tail, where the SEI is used to quantify persistent scar hypertrophy, standard rat dorsal excisional wounds rarely develop hypertrophic scars [42,43,44,45]. The NTI is therefore not intended to quantify scar hypertrophy but rather to provide a comparative estimate of overall repair-tissue thickness relative to adjacent normal skin. This internal-reference approach was intended to provide a standardized morphometric estimate of overall repair-tissue thickness after wound closure. An NTI of 100% indicates repair tissue thickness comparable with adjacent normal skin, whereas lower values indicate thinner repair tissue. In this study, there were no significant differences between the groups, although the *LD* and *LS* groups exhibited NTI values close to the adjacent normal skin thickness (*LD* ≈ 99%, *LS* ≈ 100%), while lower values were observed in the CACS and SOC groups (≈87% and ≈81%, respectively). The NTI should therefore be interpreted as informative but could, in future research, be complementary as neo-tissue structural information along with wound-healing endpoints, epithelial continuity, collagen deposition and fibroblast infiltration.

The absence of significant differences in revascularization and several other histological outcomes may reflect true comparability between treatments but may also partly reflect the small sample size, restricted scoring range, relatively small wounds, and short follow-up period. These non-significant findings and numerical trends should therefore be interpreted cautiously.

Fibroblasts are among the key cells that migrate to the wound site during the proliferative phase of healing, where they contribute to extracellular matrix deposition and the formation of granulation tissue [7]. In this study, the most prominent cellular difference detected was early fibroblast infiltration on day 7 in the *LD* group in comparison to CACS, whereas by days 11 and 14, the fibroblast scores converged across the groups. This pattern suggests that *LD* may influence early cellular dynamics of repair while later phases of healing proceed similarly across treatments within the timeframe studied. Quantitative collagen analysis differentiated the treatment groups at later timepoints. Although the collagen area fraction did not differ significantly between the groups on day 11, by day 14, the *LD* group showed a significantly higher collagen area fraction than *LS*, CACS, and SOC. These findings suggest a time-dependent divergence in extracellular matrix deposition in the *LD*-treated wounds. However, increased collagen area fraction alone should not be interpreted as evidence of improved functional regeneration, as it may also reflect greater scar matrix deposition. Additional studies evaluating collagen architecture, organization, and maturity, together with longer-term follow-up, will be required to determine whether these differences correspond to favorable dermal remodeling or increased fibrosis.

Overall, these findings suggest that the *LD* and *LS* scaffolds influenced early wound healing and tissue restoration within the 14-day study period. Although all groups ultimately achieved wound closure by day 14, the *LD* scaffold was associated with more favorable early repair dynamics, reflected by faster wound closure, earlier fibroblast infiltration, and increased collagen deposition at the final timepoint. At the same time, the lack of significant differences in the NTI and the convergence of the fibroblast scores at later timepoints indicate that the broader course of healing remained comparable across treatments. Together, these results support the potential of scaffolds derived from seaweed, particularly *LD*, to influence early wound repair while underscoring the need for longer-term studies to determine whether the observed matrix differences reflect improved tissue remodeling or increased scar formation.

### 4.3. Interpreting Healing Differences Considering Wound Treatment Properties and Study Design

The wound-healing treatments evaluated in this study were all applied in the form of sheets. However, their microstructural properties differed considerably. The *LD* and *LS* scaffolds were essentially ECM porous structures on the micrometer scale, with mean pore sizes of 48.9 ± 77.5 µm vs. 19.95 ± 17.72 µm, respectively. In contrast, the CACS used in this study was a bacterial cellulose sheet characterized by nanoscale pores, and the Tegaderm film acted primarily as an occlusive, moisture-retentive barrier [46].

The pore dimensions of the *LD* and *LS* scaffolds may also be considered in relation to other plant-derived, animal-derived, and synthetic scaffolds. Their measured pore dimensions ranged from approximately 8.8 ± 1.8 to 162.2 ± 89.3 µm, overlapping with the dimensions reported for plant-derived scaffolds, which ranged from approximately 70 ± 12 to 420 ± 33 µm [19,47]. Reported pore sizes also include approximately 132 µm for bovine tendon collagen scaffolds, 379.2 ± 34.8 µm for porcine small-intestinal submucosa sheets, and 589 ± 297 µm for a polyurethane-based biodegradable temporizing matrix [25,48].

The *LD* and *LS* scaffolds exhibited natural heterogeneous and hierarchical pore architectures. Their smaller surface chambers, close to the surface, approximately 20–50 µm, fell within a microporous range reported to support cell attachment and migration, whereas the larger interconnected tunnels, approximately 55–80 µm, may have facilitated deeper cellular infiltration, nutrient transport, vascular ingrowth, and extracellular-matrix deposition [49,50,51,52]. Previous characterization has also demonstrated larger and more heterogeneous internal features in *LD* than in *LS* [24]. These differences may have contributed to the greater early fibroblast infiltration and higher collagen area fraction observed in the *LD* group. However, re-vascularization did not differ significantly among the treatment groups, and the present study did not directly assess cellular penetration or vascular ingrowth within the scaffolds. Scaffold performance therefore cannot be attributed to pore size alone but likely reflects the combined influence of pore architecture, interconnectivity, porosity, composition, hydration, surface chemistry, thickness, and degradation. Consequently, a direct causal relationship between pore architecture and the observed wound-healing outcomes cannot be confirmed.

The production method used for the *LD* and *LS* scaffolds should also be considered when interpreting their biological performance. The visible-light-based process was designed to remove cellular material while preserving the native three-dimensional architecture of the macroalgal matrix [24]. Sequential exposure to visible light under continuous cold-water flow and aeration was followed by lyophilization and vaporized hydrogen peroxide sterilization. This method difference formed approaches where individual seaweed polymers were extracted and subsequently reformulated. The visible light-method retained an intact polysaccharide-rich structure containing cellulose, alginate, fucoidan, and other residual matrix constituents [24]. Preservation of the native architecture may therefore provide both structural and fluid-handling properties relevant to wound repair. However, lyophilization and vaporized hydrogen peroxide sterilization may alter the scaffold microstructures, surface chemistry, or biological activity of retained components. The findings observed in this study therefore cannot be attributed to a single structural or biochemical feature but likely reflect the combined effects of scaffold architecture, composition, hydration properties, and processing methods.

Beyond the scaffold’s architecture, polysaccharides found in seaweed have been investigated for wound-healing activity in vivo [53]. For instance, fucoidan has been evaluated in full-thickness rat excisional wounds, with reports of accelerated closure and changes in matrix outcomes compared to control methods [53]. Similarly, alginate-based hydrogels are widely used as wound dressings and have been shown to improve in wound healing in rat wound healing models [21,54]. Alginate dressings can absorb approximately o 15–20 times their original weight, whereas the *LD* and *LS* scaffolds showed absorption capacity within a comparable range of approximately 10-15 times their orginal weight [24,55]. In wound care, absorption and moisture retention properties of the wound care treatment are important to maintain appropriate moisture levels in wounds. Correct moisture in wounds will accelerate healing, diminish discomfort, and enhance cosmetic outcomes [56].

BC dressings are frequently reported to support wound healing in animal models, largely attributed to high water retention, conformability, and barrier properties [57]. BC-based composite dressings have also shown improved closure and histological repair in full-thickness rat wound models in multiple studies [53]. In the present study, the specific CACS comparator showed a slower wound closure rate than the *LD* and *LS* scaffolds at the first two timepoints and slower initial fibroblast infiltration. This discrepancy does not necessarily contradict the broader BC literature, as outcomes vary with BC type, dressing thickness, wound environment, and animal model [57].

The wound size and within-animal study design should also be considered when interpreting both the wound-closure and histological findings. Four 8 mm wounds were created on each rat so that every animal received all four treatments, allowing each rat to serve as its own biological control. This design reduced inter-animal variability and limited the number of animals required. However, the relatively small wound size and a 14-day follow-up period did not capture later scar maturation and remodeling. Splint displacement and periods of un-splinted wounds may have introduced variability in the contraction and closure measurements. The rapid healing capacity of young rats likely contributed to the near-complete wound closure observed in all groups by day 14. This convergence reduced the discriminatory capacity of the model at the final timepoint. A larger single wound per animal might have prolonged healing and increased the opportunity to detect treatment-related differences, although this would have required a parallel-group design and likely a greater number of animals.

Future studies should therefore include larger cohorts, longer follow-up periods, and quantitative assessment of residual scaffold material to define the degradation profiles of both scaffolds. Additional analyses should include quantitative histomorphometry and immunohistochemical assessment of repair mechanisms, including CD31-positive vessel density, to investigate the numerical trends observed in re-vascularization. Collagen deposition, fiber organization, and matrix maturation should be evaluated over longer follow-up periods to determine whether repair tissue develops toward regenerative dermal healing or fibrotic scar formation. Functional outcomes, including epidermal barrier recovery and mechanical strength, would further clarify whether the observed histological differences translate into clinically meaningful tissue restoration. Further physicochemical characterization should also assess water-retention capacity and the biodegradation of the *LD* and *LS* scaffolds in vivo.

## 5. Conclusions

In conclusion, brown macroalgal scaffolds derived from *LD* and *LS* were non-cytotoxic, well-tolerated in vivo, and compatible with normal wound repair in a splinted full-thickness rat wound model. Although all treatment groups achieved near-complete wound closure by day 14, the macroalgal scaffolds, particularly *LD*, were associated with faster intermediate wound closure relative to CACS, enhanced early fibroblast infiltration, and higher collagen area fractions at the final timepoint. At the same time, inflammation, foreign-body response, and revascularization remained broadly comparable across groups. Together, these findings support further investigation of structurally intact macroalgal matrices as sustainable regenerative biomaterials for wound healing.

## Figures and Tables

**Table 1:** Key previously reported characteristics and mechanical properties of *LD* and *LS* scaffolds relevant to the present wound-healing study. Values are summarized from a previously published evaluation [24].

Properties	*LD* Scaffold	*LS* Scaffold
Decellularized ^1^	No visible cells/nuclei	No visible cells/nuclei
Average pore size ^2^	48.9 ± 77.5 µm	19.95 ± 17.72 µm
Tunnel diameter ^3^	81 ± 62 µm	55 ± 24 µm
Porosity	67.37 ± 12.02%	67.63 ± 12.22%
Absorption after 24 h	1050.7% weight gain	1494.3% weight gain
Ultimate tensile strength	8.73 ± 0.98 MPa	6.61 ± 2.16 MPa
Young’s modulus	19.19 ± 5.51 MPa	16.19 ± 4.94 MPa

^1^ Histological evaluation via light microscope of H&E stain scaffold samples, ^2^ SEM, ^3^ Micro-CT.

## Data Availability

The raw data supporting the conclusions of this article will be made available by the authors on request.

## Abbreviations

The following abbreviations are used in this manuscript:

LD	Laminaria digitata
LS	Saccharina latissimi (Laminaria saccharina)
CACS	Commercially available cellulose sheet (EPIPROTECT^®^)
SOC	Standard of care (Tegaderm)
BC	Bacterial cellulose
NTI	Neo-tissue index
SEI	Scar elevation index
Micro-CT	Micro-computed tomography
H&E	Hematoxylin and eosin staining
AB	alamarBlue
MT	Masson trichrome staining
